# An Account of the Practice of One of the Physicians of the Westminster General Dispensary, and of the Western Dispensary, from the 20th of June, to the 20th of July, 1807

**Published:** 1807-08

**Authors:** Samuel Fothergill

**Affiliations:** Southampton Street, Strand


*An Account of the Practice of one of the Physicians of the Westminster General Dispensary, and of the Western Dispensary, from the 20th of June, to the 20th of July,* 1807.

| | | | |
|---|---|---|---|
| ACUTE DISEASES. | | Small-pox | 1 |
| Fever | 8 | Acute Diseases of Infants | 5 |
| Catarrh | 3 | Tic Douloureux | 1 |
| Acute Rheumatism | 2 | Quotidian | 2 |
| Pleurisy | 1 | Tertian | 1 |
| Erysipelas | 1 | CHRONIC DISEASES. | |
| Inflammatory Sore Throat | 1 | Asthenia | 18 |

Cough

| | | | |
|---|---|---|---|
| Cough and Dyspnœa - | 12 | Anasarca - - | 4 |
| Pulmonary Consumption | 5 | Paralysis - - - | 2 |
| Hæmoptoe - - | 2 | Lumbago - - - | 6 |
| Diarrhœa - - - | 8 | Chronic Rheumatism | 4 |
| Bilious Vomiting - | 2 | Hypochondriasis - | 2 |
| Dyspepsia - - - | 4 | Ulcerated Palate - | 1 |
| Hæmatemesis - - | 1 | Itch - - - - | 3 |
| Hæmorrhoids - - | 1 | Scabies - - - | 1 |
| Gastrodynia - - | 5 | Lepra - - - | 1 |
| Worms - - - | 2 | Chlorosis - - - | 2 |
| Rickets - - - | 1 | Amenorrhœa - - | 4 |
| Cephalalgia and Vertigo | 7 | Menorrhagia - - | 5 |
| Hydrocephalus Internus | 1 | Dysmenorrhœa - | 1 |
| Ascites - - - | 1 | Leucorrhœa - - | 4 |

In giving a monthly Report of the diseases which fall under our immediate notice, in so short an interval of time, it must frequently happen, that no case shall occur, where practical remarks or novel observations can be made; and that no important or interesting complaints have prevailed. To those, however, who wish to compare the diseases which appear in this large metropolis from year to year, and from month to month, even the list of names will be interesting; for by comparing several of these lists made by different practitioners, an estimate may be formed of the general state of disease at any particular period. From the present Report, it will be seen that the number of diseases is diminished, and that no contagious or epidemic disorders prevail within the district specified. The cases referred to the head *Fever*, were slight; some of them were the common summer fever, or synochus; whilst the symptoms of the others were too mild to be designated by that term, which, though implying a disease less severe than typhus, yet gives us to expect more serious indisposition than that to which I allude.

The cases of Asthenia are numerous, and seem to be increasing. It may be proper to state, that under this head, I class those cases of debility, and nervous disorders, which cannot be referred to any specific disease. The symptoms of this complaint are, lassitude, pain in the head, back, and loins, and frequently over the whole body when exercised; drowsiness, and aversion from any exertion, with great prostration of strength. The pulse is feeble and frequently quicker than usual; respiration is sometimes difficult, and quick; not from any defect of the lungs, lungs, but from deficiency of muscular power. The patient loses his appetite, and when food is taken, indigestion often follows. Dr. Willan, in his account of this complaint, has observed, "There is a faintness, or depression, referred to the stomach, which calls for a frequent supply of nourishment; but, as the craving is not seconded by a proportionate activity of the digestive powers, an overcharge soon takes place, and produces heart-burn, flatulency, violent pains of the stomach, or nausea, with bilious vomitings, and diarrhœa." These, however, are more frequently the characteristics of dyspepsia than of asthenia, in which they are not always present.

These symptoms are sometimes accompanied with despondency; dislike to accustomed pursuits; restlessness, and peevishness; the most gloomy ideas distress the mind and disturb the judgment; the action of the heart is affected, and palpitation and irregular pulsation, frequently alarm the patient; whilst disturbed sleep and unpleasant dreams, increase his general debility. This complaint, sometimes, is the consequence of preceding disorders; when this is not the case, it seems to be brought on by long continued fatigue; anxiety; disappointment; ennui; sedentary and unvarying occupations; hot weather; and living in close apartments without a free circulation of air. The most obstinate cases are in men who have laboured hard, and prematurely exhausted their vigour; and those who have lived temperately, and have thereby escaped dropsy and diseased livers, at the age of forty or fifty, are often the subjects of asthenia. Intemperance of every description also lays the foundation for it. Women soon after the cessation of the catamenia are frequently subject to this complaint; as also are men, who having led an active life, suddenly retire from their accustomed occupations; in such cases it is sometimes the precursor of hypochondriasis and insanity; and is preceded by ennui, which in men of a sanguine melancholy temperament, should be most resolutely combated on its first approach. Artists, and literary men, are very liable to asthenia; they labour with eagerness and alacrity, whilst any prospect of success opens before them; but often their exertions fail, and melancholy, anxiety, and despondency benumb their souls, and enfeeble their frame. Nothing is more debilitating than fruitless mental exercitation, attempting celebrity and not attaining it.

With respect to the treatment, little need be said; the principal thing is to ascertain the cause of the disease; which which in people above the lower rank, will very frequently be found to be moral, and to require a considerable change in their mode of thinking; diversity of scene and the pursuit of every thing which can afford pleasing and cheerful ideas. The medicines should be of the tonic stimulating kind; and sea-bathing is highly beneficial; where that cannot be resorted to, the cold bath may be substituted with advantage.

SAMUEL FOTHERGILL.

*Southampton Street, Strand, July* 23, 1807.

